# Light Irradiation Coupled with Exogenous Metal Ions to Enhance Exopolysaccharide Synthesis from *Agaricus sinodeliciosus* ZJU-TP-08 in Liquid Fermentation

**DOI:** 10.3390/jof7110992

**Published:** 2021-11-20

**Authors:** Hongyun Lu, Siyu Liu, Shengliang Zhang, Qihe Chen

**Affiliations:** Department of Food Science and Nutrition, Zhejiang University, Hangzhou 310058, China; luhongyun@zju.edu.cn (H.L.); 22013079@zju.edu.cn (S.L.); 12113064@zju.edu.cn (S.Z.)

**Keywords:** *Agaricus sinodeliciosus* var. *Chaidam* ZJU-TP-08, light irradiation, extracellular polysaccharide, metal ions, oxidative stress

## Abstract

To promote *Agaricus sinodeliciosus* var. *Chaidam* ZJU-TP-08 growth and metabolites accumulation, a novel integrated strategy was developed by adopting high levels of metal ions coupled with light treatment. The results revealed that yellow and blue light could significantly promote biomass and exopolysaccharides production, respectively. Furthermore, the yellow–blue light shift strategy could stimulate exopolysaccharides formation. Ca^2+^ ions coupled with blue light mostly promoted exopolysaccharides production related to oxidative stress, which was 42.00% and 58.26% higher than that of Ca^2+^ ions coupled with the non-light and dark cultivation without Ca^2+^ ions in 5-L bioreactor. RNA-seq was performed to uncover the underlined molecular mechanism regulated by light-induced gene expressions in exopolysaccharides biosynthesis and oxidative stress. The findings of this work provide valuable insights into adopting metal ions coupled with the light-assisted method for the macrofungus submerged fermentation for exopolysaccharides production.

## 1. Introduction

Macrofungi growth and secondary metabolites production are affected by many factors, including temperature, pH, and growth media [1]. Some metal ions are essential to many physiological processes, such as Ca^2+^ and Mg^2+^ [2,3], which could affect fungal morphology and cell metabolism. However, in excess, all metals are deleterious to the cells because of an unbalanced oxidative state, mainly by the formation of reactive oxygen species (ROS) [4]. EPS production was a protective response to stress to survive and grow in the metal-contaminated environment [5]. Upon *Pantoea agglomerans*, Ca^2+^ stress caused a decrease in total protein content and an increase in total carbohydrate with a boost in EPS formation [5]. Thus, the performance of *P. agglomerans* under metal stress indicated a potential candidate for metal bioremediation. Generally, EPS can incorporate nearby metal cations such as Mg^2+^, Fe^3+^, Mn^2+^, and Ca^2+^, which was associated with their viscous and negatively charged characteristics [6]. Additionally, EPS produced from microorganisms could bind heavy metals and allow growth even at high metal concentrations, and thus EPS was used as a biosorbent for heavy metal removal. Heavy metal pollution is a significant problem that has become one of the most severe environmental and human health hazards. It was reported that the increased EPS production in *Cyanobacteria*. was associated with Cd(II) resistance [7]. These polysaccharides are believed to produce biofilms and protect bacterial cells from desiccation, metallic trace elements, or other environmental stresses, including host immune responses, thus enhancing the chances of colonizing a special ecological niche [8]. Thus, metal ions could potentially act as stressful culture conditions to induce enhanced production of EPS.

Light is an important signal for adaptation to daily environmental changes in nature. During decades of studies, physiological and developmental responses to light irradiation have been found in many fungal species [9,10]. Upon cell growth in light or after perception of a light pulse, macrofungi could initiate considerable adaptations in their metabolic pathways. Alterations in response to light were predominantly observed in carotenoid metabolism, polysaccharide and carbohydrate metabolism, fatty acid metabolism, nucleoside metabolism, and in the regulation of production of secondary metabolites [9]. Photoreceptors for red and blue lights were identified in fungi [11,12,13], as well as associated photoresponse phenomena such as pigmentation, circadian cycle, and fruiting body formation, secondary metabolites [14,15]. It was demonstrated that light could be used in the industrial production of *Pleurotus ostreatus* as part of the protocol for triggering *P. ostreatus* mushroom flushing [16]. Blue light has an inhibitory effect on the vegetative growth rate of monokaryons and dikaryons [16]. Additionally, different wavelengths of light were also reported to affect *Ganoderma lucidum* growth in submerged culture conditions. The findings showed that blue light enhanced production for *G. lucidum* mycelia growth, followed in order by white light, darkness, red light, and yellow light [17]. Hence, light should be an important factor in cell growth and the secondary metabolism of macrofungi.

*Agaricus sinodeliciosus* var. Chaidam (abbreviated as *A. sinodeliciosus*), a large wild underground edible mushroom growing in the Qinghai-Tibet plateau, is rich in protein, minerals, and other nutrients, as well as bioactive substances such as polysaccharides, phenolic acids, and terpenoids. In particular, polysaccharides from *A. sinodeliciosus* have anti-hypoxia efficacy; have anti-tumor, anti-virus, and anti-oxidation properties; regulate immunity; and are hypoglycemic and hypolipidemia [18,19,20,21,22], which endow this macrofungus with extremely high research value. However, due to its low yield, further application and popularization of *A. sinodeliciosus* were limited.

To date, whether the underground *A. sinodeliciosus* is susceptible to light is still unknown. To the best of our knowledge, none has focused on the effects of light irradiation coupled with metal ions on macrofungus growth or metabolites production. Herein, in combination with the growth characteristic of *A. sinodeliciosus* var. Chaidam ZJU-TP-08 in Qinghai-Tibet plateau, China, the effects of metal ion and light irradiation on mycelial growth and EPS formation were elucidated to provide a theoretical basis for the study of metal ion dependence and photosensitive characteristics in the *A. sinodeliciosus* ZJU-TP-08 fermentation culture and the acquisition of high-yielding fermentation products upon high metal ions presence with the aim of contaminated water reuse.

## 2. Materials and Methods

### 2.1. Microorganisms, Culture Media and Cultivation Conditions

The inclining strain ZJU-TP-08 of *A. sinodeliciosus* var. Chaidam preserved in the China Center for Type Culture Collection (CCTCC M 2021511) was grown in potato dextrose agar (PDA) solid slant medium, incubated at 18 °C, and maintained at 4 °C in the darkness. Then, the liquid fermentation cultures were grown in 250 mL shaker flasks containing 100 mL culture medium composed of the following ingredients (g/L): glucose 30, peptone 5, KH_2_PO_4_ 1.0, MgSO_4_·7H_2_O 0.5, and Vitamin B1 0.5. The screening fermentation was carried out in an Erlenmeyer flask with 10% inoculum on a rotary shaker at 130 rpm and 23 °C for 8 d. After screening for different metal ions and lights, the fermentation control of light shift was conducted in a 5-L bioreactor with specific light irradiation equipment on the outer surface of the bioreactor.

### 2.2. Analytical Methods of Mycelial Biomass and EPS

After fermentation, mycelia were filtered and washed three times with distilled water to remove medium components before freeze-drying to a constant weight. The mycelium biomass (BIO) was determined as the index of mycelial growth. After filtering, 5 mL fermentation broth was added to 95% (*v*/*v*) ethanol and shaken well for 24 h at 4 °C before centrifugation. Ethanol was rinsed and freeze-dried. The content of EPS was assayed by the phenol-sulfuric acid method, as previously reported [22]. The calibration curve of EPS was generated by linear regression of OD_490_ value against glucose concentrations [23]. The calibration curve showed good linearity between OD_490_ (y) against the reduced sugar (x, µg/mL) over the calibration ranges (y = 0.0044*x* − 0.0088, R^2^ = 0.999).

### 2.3. Detection of Intracellular Reactive Oxygen Species (ROS)

The intracellular ROS detection was referred to Eruslanov [24] with minor modifications. In brief, after the fermentation broth was treated with light, 2 mL fermentation broth was centrifuged. Then, the precipitate was washed with 1 × PBS three times. Finally, 0.5 mL 1 × PBS was added to obtain the mycelia collection solution. An amount of 1 µL of 10 mM fluorescent probe DCFH-DA (Beyotime Biotechnology Co., Ltd., Haimen, China) was added and incubated in the darkness for 0.5 h at 30 °C. Mycelia were washed with PBS three times. Mycelial ROS was detected by laser scanning confocal microscope at the excitation wavelength of 488 nm and emission wavelength of 525 nm. Additionally, ultrasonic crushing was performed on the recovered pellets at 4 °C. The ultrasonic crushing power was 500 W. Every ultrasonic crushing 2 s was rest for 2 s, the total ultrasonic crushing time was 5 min, then centrifugation, the upper suspension was extracted, and the upper suspension was determined by fluorescence spectrophotometer intensity of DCF in the culture liquid to quantitatively detect the content of mycelial ROS (excitation wavelength 485 nm, emission wavelength 525 nm).

### 2.4. Measurement of Superoxide Anion (O_2−_) and Hydrogen Peroxide (H_2_O_2_)

The detection methods referred to Wu [25] and were minor modified. The mycelia of *A. sinodeliciosus* were filtered and cleaned with water three times and then filtrated. After removing the mycelia water with absorbent paper, 0.2 g of wet mycelia were weighed and added into 1.5 mL PBS (pH 7.4). Then the supernatant was centrifuged, and the content of O_2_^−^ and H_2_O_2_ was detected according to assay kits (Nanjing Jiancheng, Nanjing, China).

### 2.5. Antioxidant Enzymes and Malonaldehyde (MDA) Assay

The cultured mycelia were separated from the fermentation broth according to the above methods, and the cells were broken at 4 °C after adding 1.5 mL PBS. The supernatant obtained after centrifugation was regraded as a crude enzyme solution. Superoxide dismutase (SOD), catalase (CAT), and a glutathione peroxidase (GPx) detection kit (Nanjing jiancheng Co., Ltd., Nanjing, China) were used to detect SOD, CAT, and GPx activity. The protein concentration was measured by the protein detection kit (Sangon Co., Ltd., Shanghai China). An MDA test was also performed in strict accordance with kit instructions (Nanjing Jiancheng Co., Ltd., Nanjing, China).

### 2.6. Transcriptomic Analysis

Total RNA was extracted using Total RNA Miniprep Kit (Axygen, Hangzhou, China) and evaluated for integrity and quality using an Agilent 2100 Bioanalyzer (Agilent Technologies Inc., Amstelveen, The Netherlands)). RNA quantification was determined using the ND-2000 spectrophotometer (Thermo Inc., Wilmington, DE, USA). To analyze the transcriptome, RNA-Seq libraries were prepared from cDNA by Majorbio (Shanghai, China) and sequenced on an Illumina Hiseq4000 (Illumina Inc., San Diego, CA, USA). The raw sequencing reads were screened with the SEQPREP software and SICKLE software to cut out low-quality or default reads. All gained reads were assembled to contigs and singletons. The ORF prediction was carried out using the novel method TRINITY. Differentially expressed genes (DEGs) were identified by DEseq2 (padjust < 0.05 and difference multiple |FoldChange| > 2), as described earlier [26]. We defined gene function analysis and genome annotation from the assembled ORFs using gene ontology (GO) and the Kyoto encyclopedia of genes and genomes (KEGG). The raw RNA-seq data in this study were deposited in NCBI under accession number PRJNA729816 (https://www.ncbi.nlm.nih.gov/sra/PRJNA729816 (accessed on 17 May 2020)).

### 2.7. Statistical Analysis

All data were expressed as the mean ± standard deviation (SD). The experiments were performed in triplicates. Fisher’s least significance difference (LSD) test was performed using IBM SPSS Statistic 9 (IBM Corp., Armonk, NY, USA), *p* < 0.05 was considered as the significant threshold in this work.

## 3. Results

### 3.1. Effect of LED Irradiation Combined with Different Metal Ions on Mycelial Growth and EPS Production

**Figure 1:**
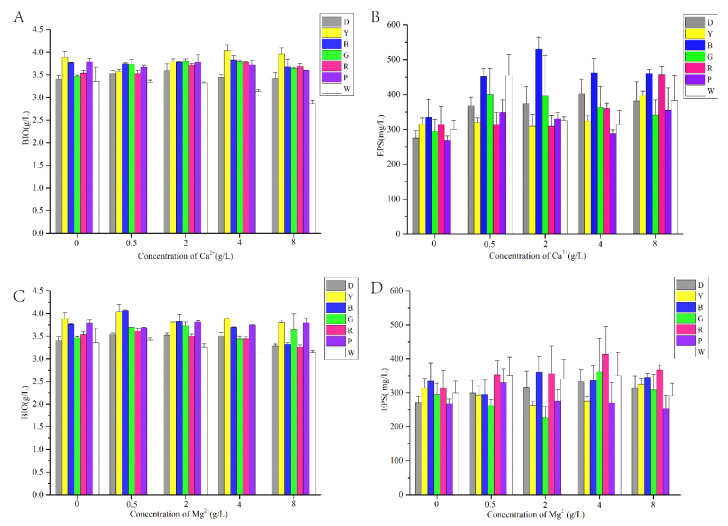
The effect of LED irradiation, including darkness (D), yellow (Y), blue (B), green (G), red (R), purple (P), and white (W) combined with metal ions on mycelial BIO (g/L) growth (**A**,**C**) and EPS production (mg/L). (**B**,**D**) *A. sinodeliciosus* var. *Chaidam* ZJU-TP-08. The concentrations of Ca^2+^ and Mg^2+^ were 0, 0.5, 2, 4, 8 g/L.

In order to detect whether the underground mushroom *A. sinodeliciosus* ZJU-TP-08 is sensitive to light irradiation and metal ions, light-emitting diodes (LEDs) with varying light wavelengths, including red, blue, yellow, purple, green, and white light, were used in the mushroom liquid fermentation. Additionally, different concentrations of calcium ion (Ca^2+^) and magnesium ion (Mg^2+^) were also added. As shown in Figure 1, by and large, LED light and metal ion stimulation play an essential role in *A. sinodeliciosus* growth. In terms of the effect of light on *A. sinodeliciosus* growth, yellow light had the most significant effect on mycelial biomass (BIO), which was 14.13% higher than that of the continuous darkness group followed by blue and purple light. With regards to EPS production, blue light was found to be optimal. Furthermore, 2 g/L CaCl_2_ coupled with blue light fermentation significantly improved EPS production, which was 42% higher than 2 g/L CaCl_2_ plus the darkness culture and 58.26% higher than non-metal ion combined darkness fermentation, respectively. In contrast, Mg^2+^ ion has an insignificant promoting effect. Hence, Ca^2+^ ions play beneficial roles in *A. sinodeliciosus* fermentation for EPS production.

### 3.2. Yellow–Blue LED Light Shift Cultivation Strategy

**Figure 2:**
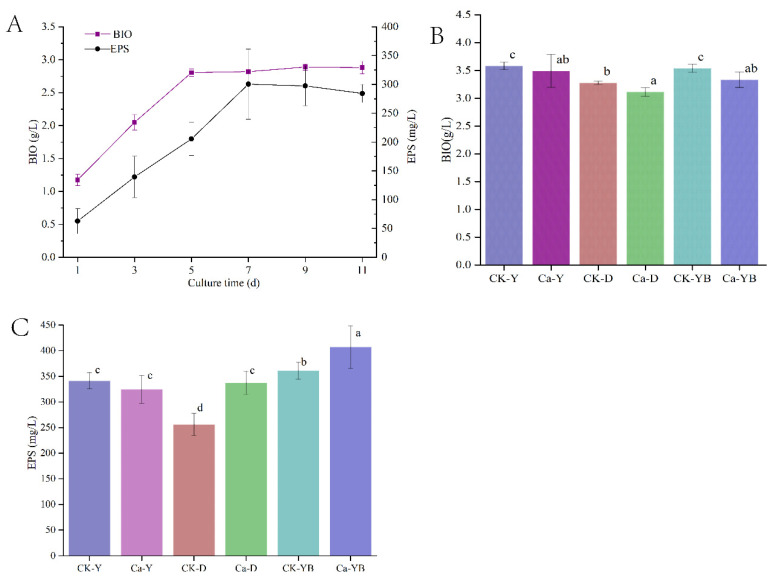
The dynamic curve (**A**) and yellow–blue LED light shift strategy of EPS synthesis (**B**) and mycelia growth (**C**). Among them, CK-Y, CK-D, and CK-YB represented the no mental ion-treated group with yellow, dark, and yellow–blue shift light, respectively. Ca-Y, Ca-D, and Ca-YB represented the Ca^2+^-treated group with yellow, dark, and yellow–blue shift light, respectively.

In order to further optimize the effect of light modulation on EPS production and mycelial growth by light irradiation, the yellow–blue LED light shift strategy was carried out in *A. sinodeliciosus* cultivation. The result of dynamically detecting the changes in the *A. sinodeliciosus* growth curve is shown in Figure 2A. In the culture stage from 0 to 11 d, the mycelia BIO and EPS synthesis curve both showed a trend of first rising and then flattening. Mycelia growth stabilized on the 5th day. However, the EPS continued to be synthesized and reached a maximum on day 7. To detect whether the photoperiod culture would further affect mycelia growth and EPS synthesis, we changed yellow light into blue light and cultivated *A. sinodeliciosus* for another 2 d after the 5th day of LED-irradiated fermentation culture. Figure 2B, 2C shows that EPS and BIO accumulation were affected by changing light wavelength during the growth stage. Our findings presented that the yellow–blue LED light shift strategy had no effect on mycelium biomass; however, it could significantly promote the synthesis of EPS. Moreover, adding 2 g/L Ca^2+^ into *A. sinodeliciosus* fermentation with yellow–blue LED light shift strategy had significant overproduced EPS yield increased by 25.48% and 59.05%, compared with yellow-light irradiation (Ca-Y) and the control (CK-D), respectively.

### 3.3. Effect of Different Cultivation Strategies on ROS, H_2_O_2_ and O_2_^−^ Contents

**Figure 3:**
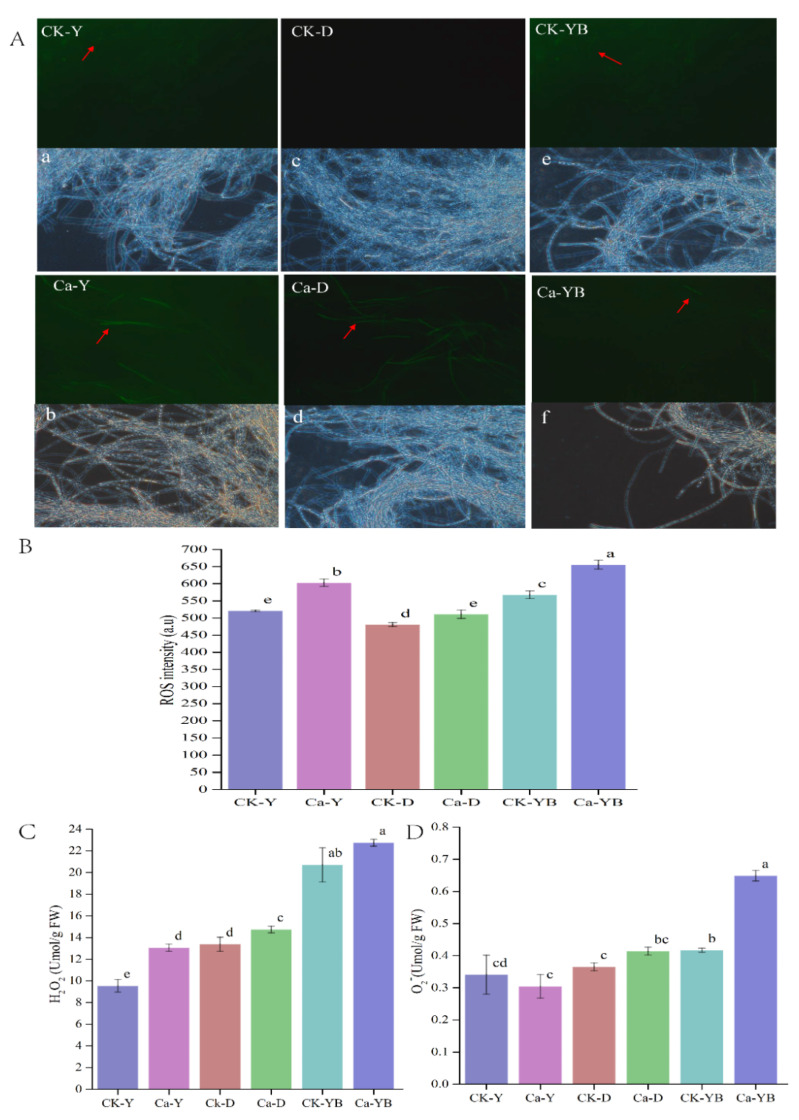
(**A**) ROS, H_2_O_2,_ and O_2_^−^ contents detection of the mycelia of *A. sinodeliciosus* var. Chaidam ZJU-TP-08 in yellow–blue LED light shift strategy. Among them (**B**,**C**), CK-Y, CK-D, and CK-YB represented the no mental ion-treated group with yellow, dark, and yellow–blue shift light, respectively. Ca-Y, Ca-D, and Ca-YB represented the Ca^2+^-treated group with yellow, dark, and yellow–blue shift light, respectively. In (**A**), images a–f represent the bright field figure for the corresponding group (above).

In order to uncover the underlined mechanism of Ca^2+^ ions and LED-light on *A. sinodeliciosus* growth and EPS production, we detected that the produced mycelial ROS in the different group, as shown in Figure 3A,B, including 2 g/L Ca^2+^ and no-mental ion (CK) coupled with darkness (Ca-D or CK-D), yellow (Ca-Y or CK-Y), yellow–blue shift (Ca-YB or CK-YB), by fluorescent probe DCFH-DA via laser confocal microscope, and fluorescence spectrophotometer. The results in Figure 3A revealed that in Ca-YB, Ca-Y, Ca-D, Ck-YB, and Ck-Y groups, the ROS fluorescence intensity was significantly detected. Additionally, the ROS in CK-D was comparatively low. Figure 3B showed the quantitative evaluation of mycelial ROS intensity in foresaid groups. In general, the ROS were produced in all groups, which might contribute to the produced oxidative stress by agitation [27]. Similarly, the ROS intensity in 2 g/L Ca^2+^ groups exposed with different LED lights, namely Ca-Y, Ca-D, and Ca-YB, had relatively higher ROS fluorescence intensity. Therefore, compared to ROS in CK-D, CK-Y, and CK-YB groups, it can be concluded that exposing LED-light in *A. sinodeliciosus* fermentation can stimulate mycelial ROS production. Accordingly, it can be concluded that LED-light exposure will promote ROS production. Besides, adding Ca^2+^ into *A. sinodeliciosus* fermentation also could increase ROS formation. As shown in Figure 3C,D, compared with the Ca-Y group, the transmission of yellow light to blue light during the culture process could significantly improve H_2_O_2_ and O_2_^−^, aggravating the oxidative stresses.

### 3.4. Detection of Glutathione Peroxidase (GPx), Superoxide Dismutase (SOD) and Catalase (CAT) Activity and Lipid Oxidation of Malondialdehyde (MDA)

**Figure 4:**
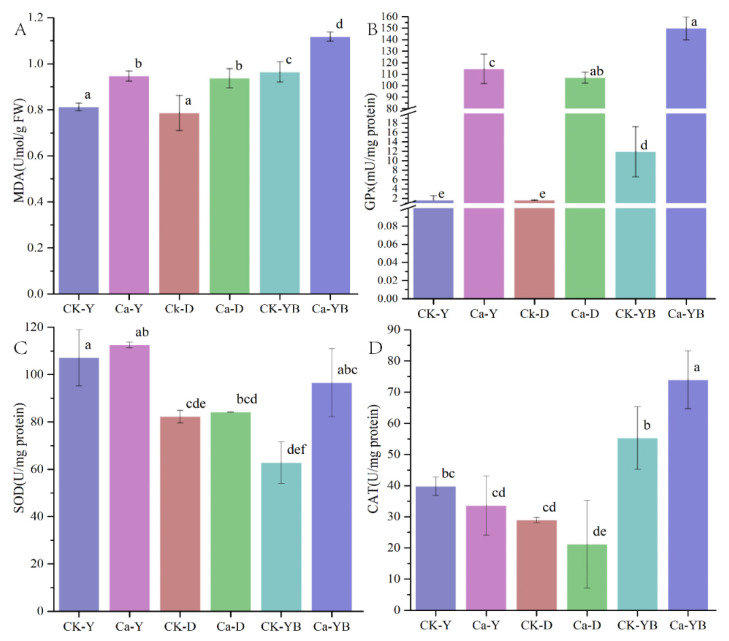
Detection of MDA (**A**), GPx (**B**), SOD (**C**), and CAT activity (**D**). CK-Y, CK-D, and CK-YB represented the no mental ion-treated group with yellow, dark, and yellow–blue shift light, respectively. Ca-Y, Ca-D, and Ca-YB represented the Ca^2+^-treated group with yellow, dark, and yellow–blue shift light, respectively.

To further investigate the effect of metal ions and light treatment on *A. sinodeliciosus* oxidative stress, the activities of SOD, CAT, GPXs, and MDA were also examined. Data from Figure 4 showed that compared with the Ca-Y group, the activities of CAT, SOD, and GPx in the culture process could be significantly increased by converting yellow–blue shift light, which could be related to the content of ROS. While light irradiation and 2 g/L Ca^2+^ could increase MDA content to different degrees; thus, oxidative stress was possibly produced.

### 3.5. Differentially Expressed Genes under Ca^2+^ and LED-Light Exposure

In order to identify genes that displayed significant changes in expression during Ca^2+^ and LED-light exposure, RNA-seq was used to investigate *A. sinodeliciosus* in response to Ca^2+^ and LED-light. After quality evaluation and trimming, over 51.07 million trimmed reads and 7.59 billion bases on average were generated from each sample under darkness conditions and LED-light conditions. All Q30 percentages for the sequences for each library were over 95%. Additionally, over 85.37% of sequenced data was mapped to assemble transcripts from the NCBI database shown in Table 1. In summary, our sequencing data were of high quality and displayed good correlations among biological replicates and thus could be used for subsequent analyses.

**Figure 5:**
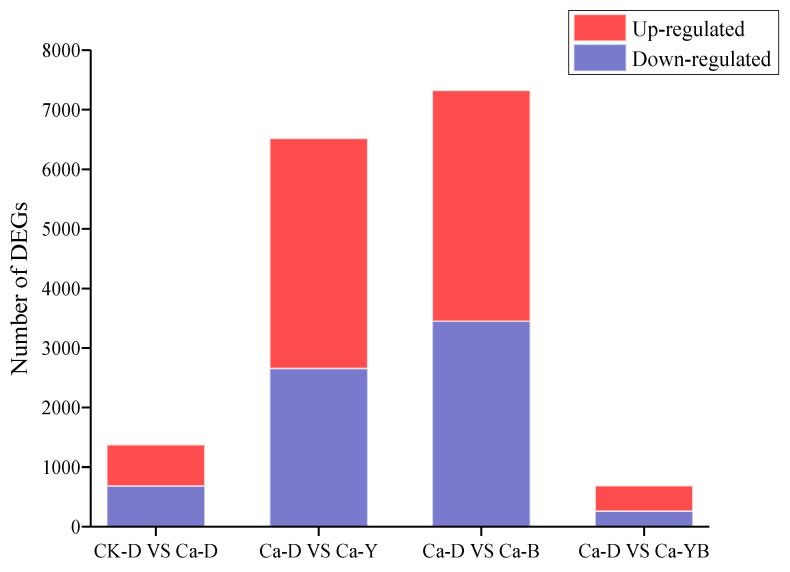
Number of differentially expressed genes with two-fold cut-off on fold changes. The X-axis shows the comparison group, including CK-D vs. Ca-D, Ca-D vs. Ca-Y, Ca-D vs. Ca-B, and Ca-D vs. Ca-YB. The Y-axis shows the number of genes significantly differentially expressed in each comparison group. The red represents up-regulated, and the blue represents down-regulated.

Accordingly, a total of 13,894 genes of *A. sinodeliciosus* were used for aligning the Illumina raw reads. Among them, the tested *A. sinodeliciosus* groups have a total of 7421 shared genes, as well as a different number of unique genes (data are shown in Supplementary Figure S1), suggesting that these genes were closely associated with *A. sinodeliciosus* growth and metabolism induced by different light qualities. We found a high number of unigenes with differential expression in treated samples. Differentially expressed genes (DEGs) with a *p* value-adjusted < 0.05 cut-off were analyzed by comparing the treated and control libraries. As can be seen from Figure 5, the resulting gene expression profiles of the control and treated samples were highly divergent. We discovered 197, 2436, and 2924 genes when comparing Ca-D versus Ca-YB, Ca-Y, and Ca-B, respectively. A total of 57 genes and 140 genes were significantly up-regulated and down-regulated in response to yellow–blue shift strategy. Moreover, after yellow light stimulation, 1128 genes and 1308 genes were significantly up-regulated and downregulated in *A. sinodeliciosus* mycelia. Additionally, blue light also had a profound effect on DEGs with 1342 up-regulated genes and 1582 down-regulated genes. Interestingly, Ca^2+^ also affected gene expressions, which were 253 up-regulated genes and 232 down-regulated genes.

### 3.6. Functional Annotation of Differentially Expressed Genes upon LED-Light Stress

**Figure 6:**
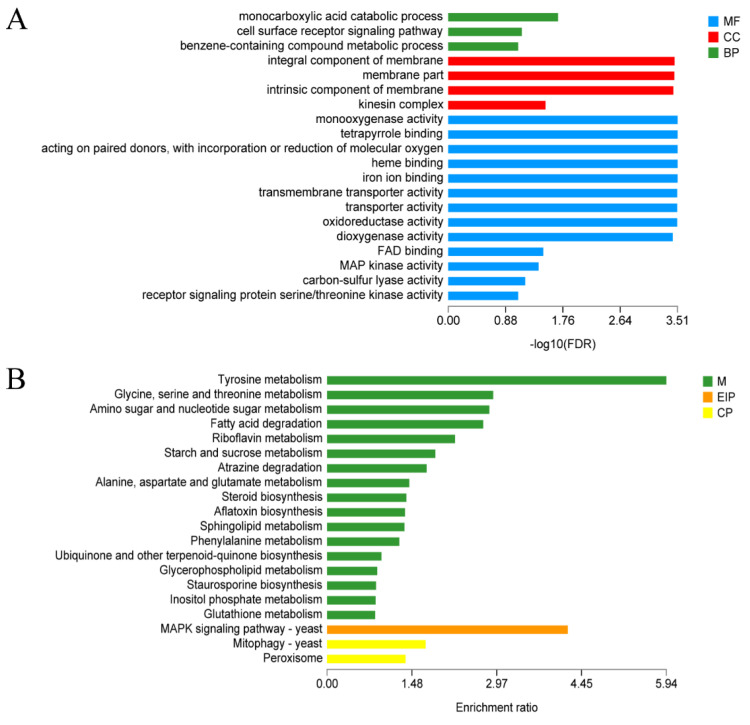
Functional annotation of DEGS in *A. sinodeliciosus* var. *Chaidam* ZJU-TP-08 under blue light (B), yellow light (Y), yellow–blue shift (YB) light stimulation. (**A**) GO annotation analysis of DEGs from Ca-D vs. Ca-Y, Ca-D vs. Ca-B and Ca-D vs. Ca-YB group. The vertical axis represents GO term, the horizontal axis represents the significance level of enrichment, and the three colors represent three categories, namely biological process (BP), cell component (CC), and molecular function (MF). (**B**) The top 20 KEGG annotation pathways from Ca-D vs. Ca-Y, Ca-D vs. Ca-B, and Ca-D vs. Ca-YB group. The vertical axis represents the KEGG pathway, and the abscess axis represents the significant level of enrichment. Different colors represent three branches of the KEGG metabolic pathway, namely cell metabolism (M), environmental information processing (EIP), and cellular process (CP).

Given that the light-controlled coupling Ca^2+^ fermentation has an important effect on the liquid fermentation of *A. sinodeliciosus*, the identified genes were annotated by GO and KEGG databases to investigate the function of up-regulated genes better. For GO enrichment analysis, the enriched annotations were grouped into three functional categories: biological processes, cellular components, and molecular functions (Figure S2). Our analysis revealed significantly enriched biological process terms (*p* < 0.05) associated with cell growth and development, such as positive regulation of metabolic process (GO: 0008152), biological regulation (GO:0065007), cellular process (GO:0009987), and response to stimulus (GO:0050896). Among them, compared with yellow light, blue light enhanced the expression of genes related to response to stimuli. Within the cellular component category, membrane part (GO:0044425), cell part (GO:0044464), and organelle (GO:0043226) were rich in DEGs. For further detecting the DEGs in *A. sinodeliciosus* response to light stimulation, GO enrichment analysis showed in Figure 6A revealed that the molecular function, ‘transmembrane transporter activity’ (GO:00022857, 378 genes), ‘transporter activity’ (GO:0005215, 381 genes), ‘oxidoreductase activity’ (GO:0016491, 501 genes), ‘iron ion binding’ (GO:0005506, 82 genes), ‘FAD binding’ (GO:0071949, 42genes), ‘catalytic activity’ (GO:0003824, 14971 genes) were abundantly enriched under blue, yellow, and yellow-shift light conditions, along with integral or intrinsic component of the membrane and cell surface receptor signaling pathway. KEGG analysis was used to classify the functions of annotated DEGs further. In addition to the pathways related to metabolisms, such as tyrosine metabolism, glycine, serine, and threonine metabolism, Figure 6B revealed that mitogen-activated protein kinase (MAPK) signaling pathways and oxidative phosphorylation were enriched in DEGs.

**Figure 7:**
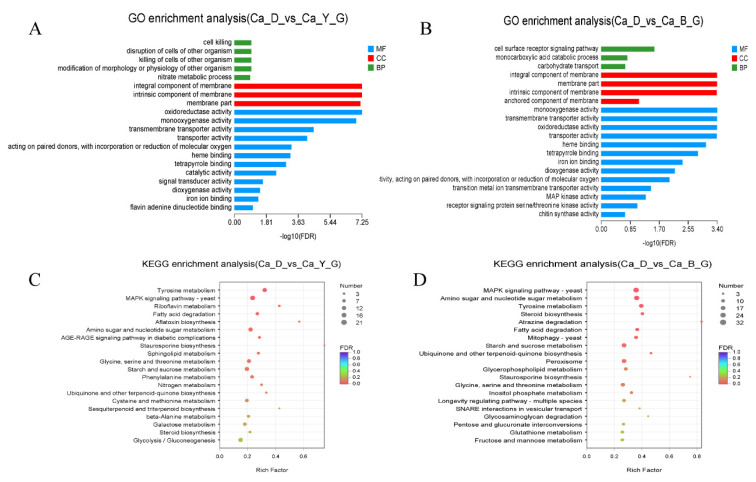
Functional annotation of DEGS in *Agaricus sinodeliciosus* var. *Chaidam* ZJU-TP-08 under blue light (B), yellow light (Y), yellow–blue shift (YB) light stimulation. (**A**,**B**) GO annotation analysis of DEGs from Ca-D vs. Ca-Y and Ca-D vs. Ca-B group. The vertical axis represents GO term, the horizontal axis represents the significance level of enrichment, and the three colors represent three categories, namely biological process (BP), cell component (CC), and molecular function (MF). (**C**,**D**) The top 20 KEGG annotation pathways from Ca-D vs. Ca-Y and Ca-D vs. Ca-B group. The vertical axis represents the KEGG pathway, and the abscess axis represents the significant level of enrichment. Different colors represent three branches of KEGG metabolic pathway, namely metabolism (M), environmental information processing (EIP), cellular process (CP).

In order to further investigate the effects of yellow (Y) and blue light (B) on liquid fermentation of *A. sinodeliciosus*, we analyzed DEGs by GO and KEGG enrichment analysis at the transcriptome level. The GO enrichment analysis shown in Figure 7A,B revealed that the DEGs of *A. sinodeliciosus* under blue-light and yellow-light stimulation were significantly enriched in membrane component and part, oxidoreductase activity, monooxygenase activity, and transmembrane transporter activity, which were related to cell composition and molecular function. As shown from the KEGG enrichment analysis in Figure 7C,D, DEGs in *A. sinodeliciosus* response to blue light and yellow light were mainly concentrated in tyrosine metabolism, MAPK signaling pathway, amino sugar and nucleotide sugar metabolism, and starch and sucrose metabolism. Additionally, compared with yellow light, DEGs of the peroxisome metabolism pathway were significantly enriched in blue-light stimulation.

**Figure 8:**
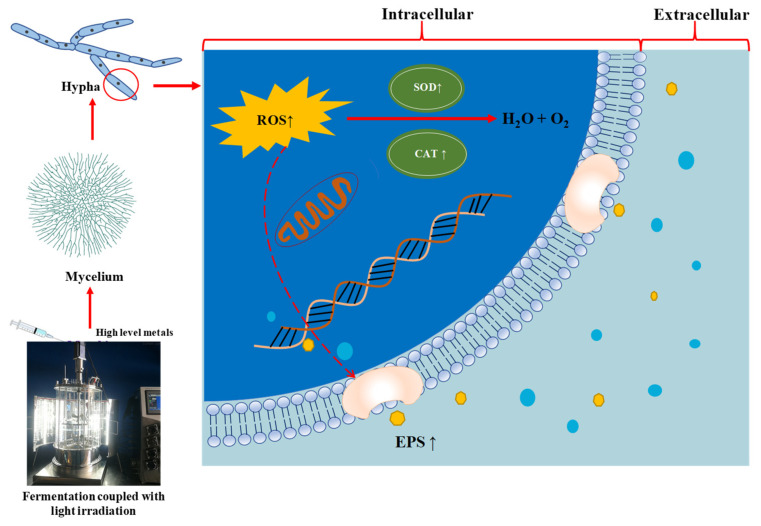
The postulated regulatory mechanism of LED-lights irridation coupled with metal ions on mycelia growth and EPS accumulation by *A. sinodeliciosus* var. *Chaidam* ZJU-TP-08.

Therefore, in order to elucidate the polysaccharide biosynthesis pathway of *A. sinodeliciosus* stimulated by blue light, we detected the up-regulated genes and gene clusters involved in the saccharide unit. The result presented in Supplementary materials Table S1 revealed that blue light could up-regulate genes related to fructose and mannose metabolism, galactose metabolism, and starch and sucrose metabolism. In this work, Table 2 revealed that blue-light stimulation led to a significant activation of the transcription of genes involved in DNA repair and cellular response to DNA damage stimulus. In addition to the above, consistent with the previous results, blue light up-regulated DEGs in peroxidase activity, catalase activity, peroxiredoxin activity, and hydrogen peroxide catabolic process to respond to oxidative stress. Hence, the transcriptomics results revealed that blue-light stimulation could provoke the antioxidative activity and stimulate EPS production by *A. sinodeliciosus*. To sum up the findings of this work, the possible mechanism of different LED lights on mycelia growth and EPS production by *A. sinodeliciosus* was postulated, which is presented in Figure 8.

## 4. Discussion

*Agaricus sinodeliciosus* var. *Chaidam*, a rare underground mushroom, has not yet been cultivated successfully. The effect of light coupled with metal ions on *A. sinodeliciosus* growth, as well as its underlying molecular mechanisms and genes, remain relatively unclear. In summary, our findings demonstrated that the addition of light exposure and metal ions could have a great effect on *Agaricus sinodeliciosus* var. *Chaidam* growth and EPS production. In our study, 2 g/L CaCl_2_ coupled with blue light fermentation significantly improved EPS production, which was 42% higher than 2 g/L CaCl_2_ plus the darkness culture and 58.26% higher than non-metal ion combined darkness fermentation, respectively. Previous research [28] demonstrated that LED illumination exhibited an adverse effect on *P. eryngii* growth but increased EPS production by blue light, which was similar to our research findings. Blue light could also be considered as the best treatment to enhance the production efficiency of oyster mushrooms [29]. Moreover, Kho [30] reported that blue LED contributed to the highest EPS production of *Cordyceps militaris*, but the red LED was the best for mycelia biomass growth, which was inconsistent with our findings. Therefore, we hypothesized that different mushrooms respond differently to light stimulation. It was reported that adding 10 mM Ca^2+^ to static liquid cultures of *Ganoderma lucidum* proved to be a useful strategy to enhance the production of ganoderic acids (GAs) [2]. Moreover, simultaneously adopting the strategies of multiple Cu^2+^ additions coupled with three-stage light irradiation could enhance total GAs produced by *G. lucidum* [31]. Therefore, the culture strategy of supplementing metal ions with light treatment is beneficial to obtain fermentation products of macrofungus. Notably, EPSs are a class of biopolymers that are secreted extracellularly by macrofungi to withstand adverse conditions [32]. Upon exposure to environmental stresses, the mushroom could secrete polysaccharides to cope with the adverse environment. Intriguingly, metal ions can stimulate polysaccharide synthesis. In metallic trace element pollution, bacterial exopolymers have become an alternative of interest as metal-binding agents in the detoxification of contaminated water [33]. It was demonstrated that Ca^2+^ could affect *Lentinus edodes* mycelial growth and polysaccharide biosynthesis [34], which was similar to the present results.

Light, as a stress factor, could induce the generation of ROS to regulate the biosynthesis of fungal metabolites [35]. Moreover, metal-induced toxicity observed in different living organisms was associated with oxidative damage [36]. ROS, including oxygen radicals and some non-radical derivatives of oxygen (O_2_), including superoxide anion (O_2_^−^) and hydrogen peroxide (H_2_O_2_), is produced as a typical cellular metabolic byproduct in living cells [37]. Therefore, O_2_^−^ and H_2_O_2_ production in the mycelia were also measured in Figure 3C,D. Ca^2+^ significantly increased the level of oxidative stress.

The effect of metal ions and light treatment on *A. sinodeliciosus* oxidative stress can also be understood by checking the oxidative system. The activities of SOD, CAT, GPXs and MDA content were also studied. SOD enzymes could control the levels of a variety of ROS, thus both limiting the potential toxicity of these molecules and controlling broad aspects of cellular life that are regulated. SOD enzymes catalyze the dismutation of superoxide (O_2_^−^), generating hydrogen peroxide (H_2_O_2_) [37]. CAT and GPXs, the key antioxidant enzymes produced to decompose H_2_O_2_ [38], were shown to be the most significantly induced antioxidant enzymes under oxidative stress in filamentous fungus [39]. Therefore, cellular H_2_O_2_ concentration and CAT activity are the appropriate indicators of oxidative stress and the fungal cellular response to it [40].

Polysaccharides are considered the major and most-studied bioactive component in macrofungus. Previous research revealed that *A. sinodeliciosus*-derived EPS is mainly composed of monosaccharides, such as xylose, arabinose, galactose, mannose, and glucose [41]. In order to investigate the EPS biosynthesis pathway of *A. sinodeliciosus*, we detected the genes and gene clusters involved in the saccharide unit, such as sucrose, mannose, and galactose biosynthesis by transcriptomics analysis. Our RNA-seq results showed that blue light could up-regulate genes related to fructose, mannose, galactose, and sucrose metabolism, which could be one reason that accounted for the increase in overproduced polysaccharide synthesis [42]. Kojima [15] demonstrated that blue light could enhance the key enzyme related to shikimic acid synthesis in submerged liquid fermentation of oyster mushroom, thus promoting the synthesis of secondary metabolite shikimic acid. Unfortunately, we did not find significant changes in key genes involved in polysaccharide synthesis, including the reported phospho-glucose isomerase (PGI), phosphoglucomutase (PGM), and UDP-glcpyrophosphorylase (UGP) [34]. We hypothesized that the increased EPS synthesis might have other pathways besides enzymes increase. Importantly, DNA was constantly exposed to damaging threats coming from photoinduced oxidative stress [43]. It was noted that blue-light stimulation led to a significant activation of the transcription of genes involved in DNA repair and cellular response to DNA damage stimulus. Threats to DNA stability can be triggered by oxidative stress induced by the presence of metabolic reactive radical and ROS or by photoreactivity, which could have a strong influence on the biological processes governed by DNA, as well as in the indirect activation or transduction of signal cascades, resulting in different outcomes for the life of involved cells [44].

## 5. Conclusions

In summary, various wavelengths of LEDs supplemented with metal ions were used to regulate mycelial biomass and EPS production in *A. sinodeliciosus* cultivation. Yellow light and blue light were the most significant promoter of mycelium and EPS production, respectively. Moreover, the combination of yellow and blue light in different fermentation stages could significantly promote the synthesis of EPS. Finally, the enhanced EPS production was obtained by blue light coupled with Ca^2+^ stimulation, which was found to attribute to oxidative stress. Our findings provided a novel cultivation strategy for high-value mushrooms to promote the production of active substances under different stresses.

## Figures and Tables

**Table 1:** The reads, bases number, and mapping rate of the *A. sinodeliciosus* transcriptome.

Sample	Raw Reads	Raw Bases	Clean Reads	Clean Bases	Q30 (%)	Mapped Ratio
CK_D1	52430690	7917034190	52004596	7722013736	95.78	88.94%
CK_D2	51419082	7764281382	51069226	7590450857	95.97	88.94%
CK_D3	55445214	8372227314	54994074	8169378821	95.97	88.81%
Ca_B1	67430876	10182062276	66924606	9809044312	95.9	88.11%
Ca_B2	57860464	8736930064	57492788	8535634916	95.65	87.00%
Ca_B3	62620904	9455756504	62213748	9201273878	95.85	87.56%
Ca_D1	61133916	9231221316	60692440	8968939360	95.81	87.08%
Ca_D2	58526628	8837520828	58136926	8614513195	95.9	87.82%
Ca_D3	63118224	9530851824	62727544	9270858115	95.95	87.60%
Ca_Y1	57675382	8708982682	57275576	8471498632	95.78	89.81%
Ca_Y2	61837888	9337521088	61473936	9120391873	95.82	87.54%
Ca_Y3	64299506	9709225406	63775712	9414716156	95.91	88.43%
Ca_YB1	64218946	9697060846	63827898	9405470492	96	85.52%
Ca_YB2	56873006	8587823906	56504146	8397225398	95.67	86.42%
Ca_YB3	53193032	8032147832	52899302	7864009316	95.83	85.37%

**Table 2:** Repression of up-regulated genes involved in oxidative stress.

Gene ID	Function	Log2fc	*p*-Value
TRINITY_DN5963_c0_g1	DNA repair	1.48	3.74 × 10^−24^
TRINITY_DN1802_c0_g1	Cellular response to DNA damage stimulus, regulation of transcription from RNA polymerase II promoter in response to stress	1.26	3 × 10^−20^
TRINITY_DN537_c0_g1	Integrin-mediated signaling pathway, DNA repair	1.07	1.74 × 10^−15^
TRINITY_DN266_c0_g1	Response to oxidative stress, metal ion binding, peroxidase activity	1.51	7.87 × 10^−26^
TRINITY_DN2103_c0_g1	Hydrogen peroxide catabolic process, response to oxidative stress, catalase activity	1.18	3.76 × 10^−10^
TRINITY_DN6330_c0_g1	DNA repair, 5′-3′ exodeoxyribonuclease activity	1.08	1.87 × 10^−8^
TRINITY_DN8497_c0_g1	Defense response to fungus, extracellular region	4.37	3.71 × 10^−14^
TRINITY_DN1858_c0_g1	DNA recombinase assembly, double-strand break repair via single-strand annealing	1.19	6.59 × 10^−9^
TRINITY_DN4463_c0_g2	integrin-mediated signaling pathway, DNA repair	1.26	8.67 × 10^−19^
TRINITY_DN4261_c0_g1	DNA repair, endonuclease activity	1.46	4.99 × 10^−17^
TRINITY_DN1061_c0_g1	DNA repair, damaged DNA binding	1.17	4.66 × 10^−17^
TRINITY_DN8232_c0_g1	Defense response to fungus	4.05	1.19 × 10^−12^
TRINITY_DN7616_c0_g1	Peroxiredoxin activity	5.56	2.1 × 10^−91^
TRINITY_DN3765_c0_g1	Integral component of membrane, peroxidase activity;	1.53	6.82 × 10^−23^
TRINITY_DN12831_c0_g1	Peroxidase activity, heme binding, oxidoreductase activity;	1.42	0.011767

## Data Availability

Sequence data used in this study were deposited at the National Center for Biotechnology Information (NCBI) as part of the BioProject PRJNA729816.

## Supplementary Materials

The following are available online at https://www.mdpi.com/article/10.3390/jof7110992/s1. Figure S1: Venn diagram between samples. Figure S2: The top 20 GO terms enriched in each category, Table S1: The up-regulate genes related to fructose and mannose metabolism, galactose metabolism, starch and sucrose metabolism by blue-light stimulation.
