# Supplementary material for: Light Irradiation Coupled with Exogenous Metal Ions to Enhance Exopolysaccharide Synthesis from Agaricus sinodeliciosus ZJU-TP-08 in Liquid Fermentation
Source: J Fungi (Basel). 2021 Nov 20;7(11):992. doi: 10.3390/jof7110992 (PMC8618256; doi:10.3390/jof7110992)
Supplement: Supplementary file 1 [file jof-07-00992-s001.zip › jof-1458685-supplementary.pdf]

Supplementary files

Light Irradiation Coupled with Exogenous Metal Ions to Enhance Exopolysaccharide Synthesis from *Agaricus sinodeliciosus* ZJU-TP-08 in Liquid Fermentation

Hongyun Lu, Siyu Liu, Shengliang Zhang and Qihe Chen *

Department of Food Science and Nutrition, Zhejiang University, Hangzhou 310058, China

* ***Corresponding author:***

Qihe Chen

Tel: +86-571-86984316

E-mail: chenqh@zju.edu.cn

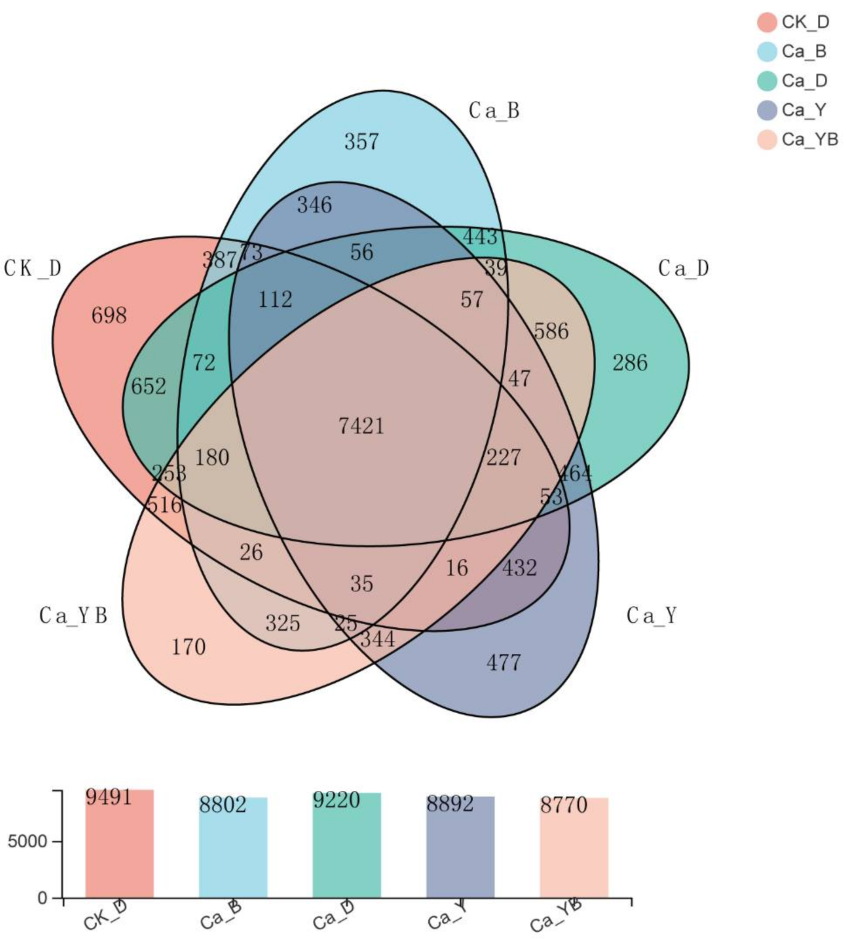

Figure.- S1. Venn diagram between samples. Different colored circles represent the number of unigene expressed in a set of samples, and the cross areas of the circles represent the number of unigene shared by each group.in the bar chart, the abscissa represents the sample name, and the ordinate represents the number of unigene.

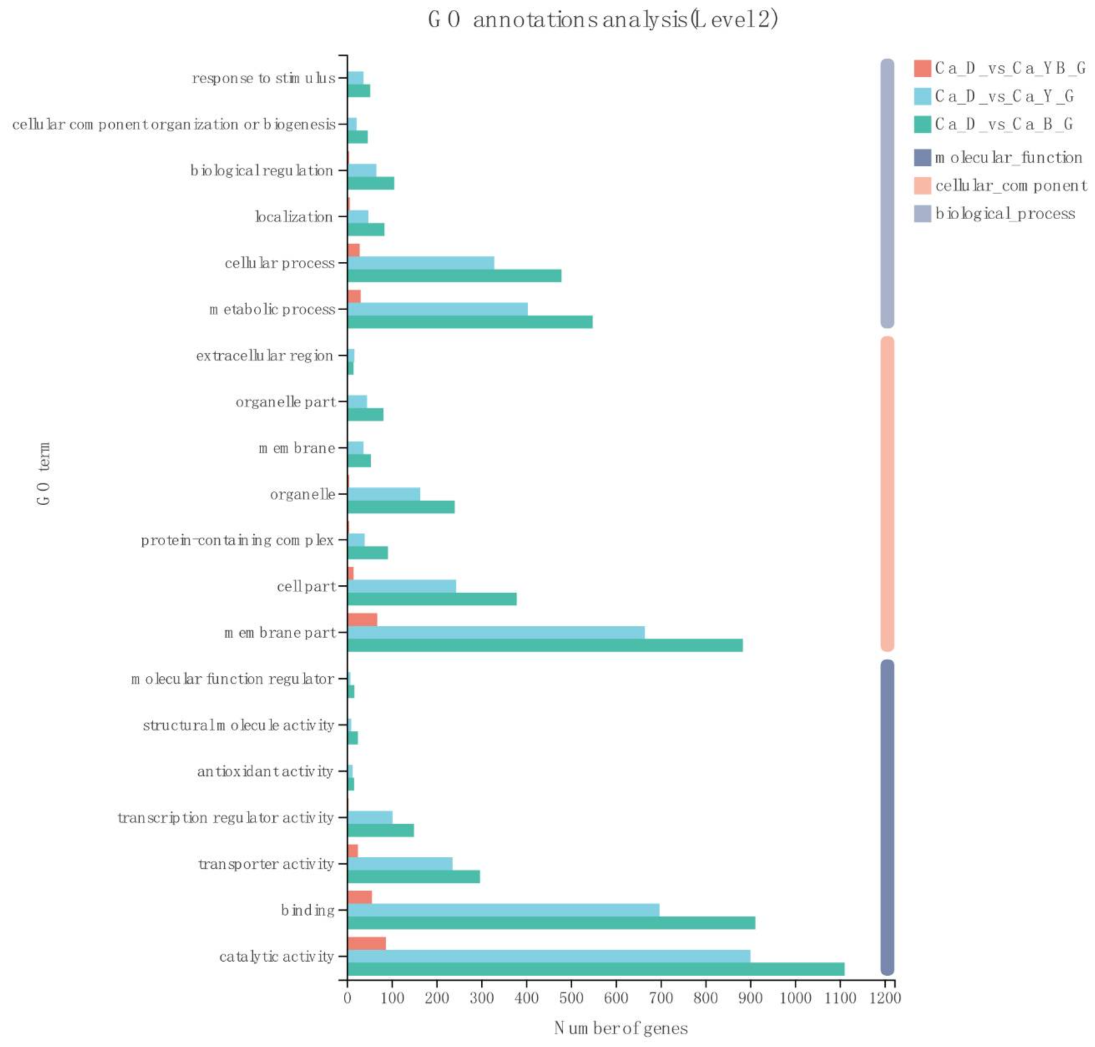

Figure.- S2. The top 20 GO terms enriched in each category (P <0.05): The left vertical axis in the figure represents the secondary classification of GO, and the abscissa represents the number of unigene contained in the secondary classification.

Table S1. The up-regulate genes related to fructose and mannose metabolism, galactose metabolism, starch and sucrose metabolism by blue-light stimulation

Gene ID	Function	Pathway definition	log2fc	Padjust
TRINITY_DN10927_c0_g1	L-rhamnonate dehydratase activity	Fructose and mannose metabolism	1.741969	5.87E-08
TRINITY_DN10882_c0_g1	cellular glucose homeostasis, glycolytic process, glucose binding	Amino sugar and nucleotide sugar metabolism, Fructose and mannose metabolism	2.34123	1.69E-59
TRINITY_DN793_c0_g1	carbohydrate metabolic process	Fructose and mannose metabolism	1.244681	4.78E-28
TRINITY_DN4143_c0_g1	GDP-mannose biosynthetic process, mannose-6-phosphate isomerase activity	Fructose and mannose metabolism	2.966049	8.36E-24
TRINITY_DN4143_c0_g1	GDP-mannose biosynthetic process, carbohydrate metabolic process, mannose-6-phosphate isomerase activity	Amino sugar and nucleotide sugar metabolism; Fructose and mannose metabolism	2.966049	8.36E-24
TRINITY_DN11724_c0_g1	carbohydrate catabolic process, carbohydrate metabolic process, alpha-amylase activity	Starch and sucrose metabolism	5.177869	6.86E-08
TRINITY_DN993_c0_g1	carbohydrate metabolic process, alpha-amylase activity, hydrolyzing O-glycosyl compounds	Starch and sucrose metabolism	2.026641	6.9E-26
TRINITY_DN10882_c0_g1	cellular glucose homeostasis, glycolytic process, glucose binding	Amino sugar and nucleotide sugar metabolism, Fructose and mannose metabolism, Galactose metabolism	2.34123	2.74E-61
TRINITY_DN4231_c0_g1	1,3-beta-D-glucan synthase complex, 1,3-beta-D-glucan synthase activity	Starch and sucrose metabolism	1.009855	4.05E-06
TRINITY_DN5101_c0_g1	hydrolase activity	Starch and sucrose metabolism	1.568751	3.07E-23
TRINITY_DN1020_c0_g1	cellulose catabolic process, carbohydrate binding	Starch and sucrose metabolism	1.684015	1.83E-16

TRINITY_DN5360_c0_g1	carbohydrate metabolic process, hydrolase activity	Starch and sucrose metabolism	1.103691	0.000665
TRINITY_DN1717_c0_g1	cellulose catabolic process, scopolin beta-glucosidase activity, beta-glucosidase activity	Starch and sucrose metabolism	1.18656	2.12E-06
TRINITY_DN5592_c0_g1	tricarboxylic acid cycle, transferring acyl groups	Citrate cycl; Glyoxylate and dicarboxylate metabolism	1.010687	4.62E-11
TRINITY_DN4524_c0_g1	oxidoreductase activity, acting on the CH-OH group of donors, NAD or NADP as acceptor;	Glyoxylate and dicarboxylate metabolism	3.648546	0.001167
TRINITY_DN3071_c0_g1	N-acetylglucosamine metabolic process, glucosamine-6-phosphate deaminase activity;	Amino sugar and nucleotide sugar metabolism	3.24014	1.13E-57
TRINITY_DN1741_c0_g1	UDP-N-acetylglucosamine biosynthetic process, tricarboxylic acid cycle, glucosamine 6-phosphate N-acetyltransferase activity;	Amino sugar and nucleotide sugar metabolism	1.957214	2.6E-15
TRINITY_DN1300_c0_g2	carbohydrate metabolic process, chitin binding	Amino sugar and nucleotide sugar metabolism	3.682973	1.78E-06
TRINITY_DN606_c0_g1	arabinan metabolic process, L-arabinose metabolic process, alpha-L-arabinofuranosidase activity	Amino sugar and nucleotide sugar metabolism	2.414531	2.89E-08
TRINITY_DN10882_c0_g1	glycolytic process, hexokinase activity	Galactose metabolism; Glycolysis / Gluconeogenesis	2.34123	1.69E-59
TRINITY_DN370_c0_g1	chitin biosynthetic process, plasma membrane	Amino sugar and nucleotide sugar metabolism	1.681899	1.33E-19
TRINITY_DN2718_c0_g1	chitin biosynthetic process, component of membrane, plasma membrane	Amino sugar and nucleotide sugar metabolism	2.186617	1.7E-29
